# Synchrotron radiation reveals the identity of the large felid from Monte Argentario (Early Pleistocene, Italy)

**DOI:** 10.1038/s41598-018-26698-6

**Published:** 2018-05-29

**Authors:** Marco Cherin, Dawid A. Iurino, Marco Zanatta, Vincent Fernandez, Alessandro Paciaroni, Caterina Petrillo, Roberto Rettori, Raffaele Sardella

**Affiliations:** 1 Department of Physics and Geology, University of Perugia, I-06123 Perugia, Italy; 2 PaleoFactory, Sapienza University of Rome, I-00185 Rome, Italy; 3 Department of Earth Sciences, Sapienza University of Rome, I-00185 Roma, Italy; 4 Department of Computer Science, University of Verona, I-37134 Verona, Italy; 5 European Synchrotron Radiation Facility (ESRF), Beamline ID19, F-38000 Grenoble, France

## Abstract

We describe here a partial skull with associated mandible of a large felid from Monte Argentario, Italy (Early Pleistocene; ~1.5 million years). Propagation x-ray phase-contrast synchrotron microtomography of the specimen, still partially embedded in the rock matrix, allows ascribing it reliably to *Acinonyx pardinensis*, one of the most intriguing extinct carnivorans of the Old World Plio-Pleistocene. The analysis of images and 3D models obtained through synchrotron microtomography – here applied for the first time on a Plio-Pleistocene carnivoran – reveals a mosaic of cheetah-like and *Panthera*-like features, with the latter justifying previous attributions of the fossil to the extinct Eurasian jaguar *Panthera gombaszoegensis*. Similarly, we reassign to *A*. *pardinensis* some other Italian materials previously referred to *P*. *gombaszoegensis* (sites of Pietrafitta and Ellera di Corciano). The recognition of *Panthera*-like characters in *A*. *pardinensis* leads to reconsidering the ecological role of this species, whose hunting strategy was likely to be different from those of the living cheetah. Furthermore, we hypothesise that the high intraspecific variation in body size in *A*. *pardinensis* can be the result of sexual dimorphism, as observed today in all large-sized felids.

## Introduction

**Figure 1:**
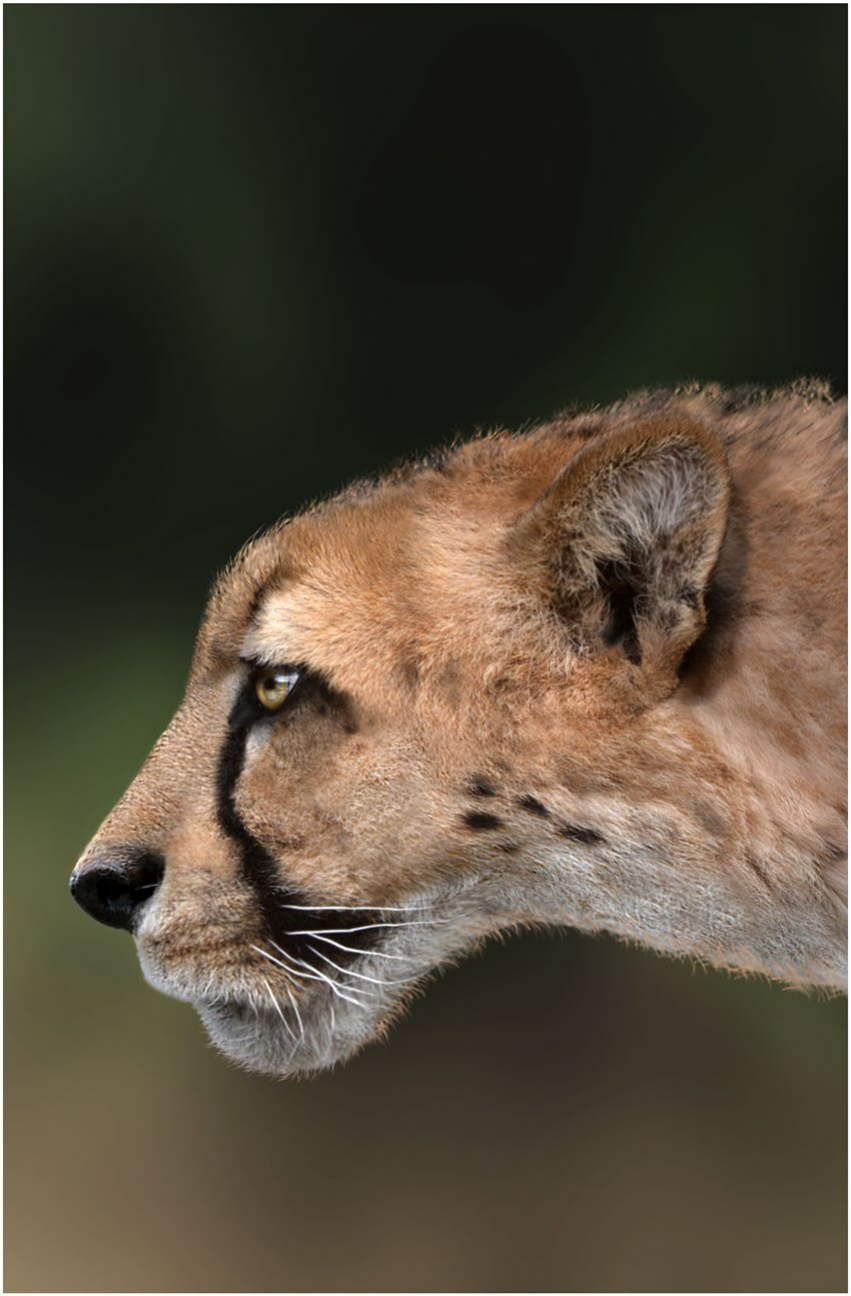
Head appearance of *Acinonyx pardinensis*. The reconstruction is based on the complete skull from Pantalla^6^ and is updated with the information on the craniodental anatomy of *A*. *pardinensis* achieved from the Monte Argentario specimen. Artwork by D. A. Iurino.

The Villafranchian Land Mammal Age (LMA) is a biochronological unit based on large mammals roughly spanning from the late Pliocene to most of the Early Pleistocene (about 3.5–1.0 million years; Ma)^1^. The mammal assemblages that characterised Villafranchian Europe were the result of the deep faunal turnover occurring between the early and late Pliocene in response to a global glaciation process involving the development of the first ice sheets in Greenland^2^. This general cooling trend coincided with the setting of the modern Mediterranean climate, resulting in significant variations in vegetation^3^ and, consequently, in the herbivore community. The latter experienced the extinction of a number of taxa adapted to the Miocene and early Pliocene warm and humid average conditions and the dispersal of several newcomers from Africa and Asia^2^. These changes deeply affected also the carnivore guild, and the large felids in particular. At the beginning of the Villafranchian LMA, at least four new large felid taxa appear: the sabretooths *Homotherium* and *Megantereon*^4^, the first member of the puma lineage *Puma pardoides*^5^, and the cheetah-like cat *Acinonyx pardinensis*^6^. The latter species (Fig. 1) is probably one of the most intriguing and iconic extinct carnivorans of the Old World Plio-Pleistocene.

The earliest fragmentary fossils of cheetah-like felids in Europe were discovered in the Puy de Dôme area, France (La Côte d’Ardé and Les Étouaires sites) in the first half of the 19th century^7,8^. However, it is necessary to wait more than a hundred years to get well-preserved cranial material, unearthed in the French site of Saint Vallier^9^. Viret^9^ acknowledged that all the remains discovered until then in Europe could be included in a single species, which he calls *Acinonyx pardinensis* in light of the many morphological affinities with the living African cheetah *Acinonyx jubatus*. To date, *A*. *pardinensis* has been reported in more than 35 locations in Eurasia and North Africa, covering a long chronological interval (about 2.5 Ma) from the late Pliocene to the Middle Pleistocene^6^. This sample includes also the Chinese specimens referred to *Sivapanthera pleistocaenicus* and *S*. *linxiaensis* by some authors^10,11^, but later recognized as belonging to *A*. *pardinensis*^6,12^.

The ecological role of *A*. *pardinensis* in the Villafranchian carnivoran guild is matter of debate. The few available postcranial material^8,13–15^ would suggest that *A*. *pardinensis* had slender body proportions similar to those of *A*. *jubatus*, though the fossil species was characterised by double body mass (~80 vs. ~40 kg on average, respectively)^6^. However, it is not possible to demonstrate whether the body structure of *A*. *pardinensis* was correlated to predatory adaptations based on high speed in open environments as in the extant cheetah^15^. On the contrary, the recent discovery of outstanding cranial material from Pantalla, Italy^6^ and the reappraisal of the specimens from Saint Vallier^12^ have led to reconsidering the skull anatomy and predatory behaviour of *A*. *pardinensis*. The craniodental morphology and the development of jaw muscles associated with the large body size, suggest that *A*. *pardinensis* could kill large prey with a hunting strategy akin to those of pantherine felids, rather than that of the cheetah^6^. This hypothesis is supported by recent analysis^16^ of the inner ear anatomy of extant and extinct felids, according to which the vestibular system of modern cheetahs is unique in showing adaptations to facilitate visual and postural stability during high-speed prey pursuits and is extremely different in shape and proportions relative to other species, including *A*. *pardinensis*.

**Figure 2:**
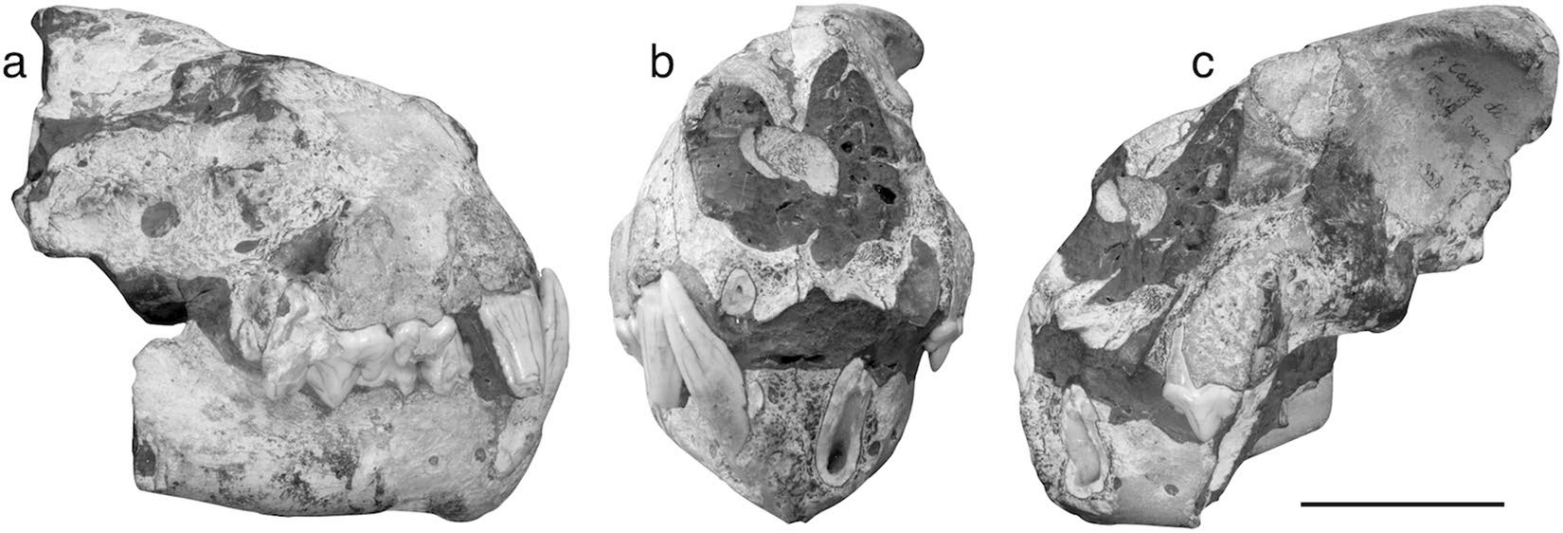
*Acinonyx pardinensis* from Monte Argentario. Specimen ArgBsc1 in right lateral (**a**), rostral (**b**), and left lateral (**c**) views. Scale bar: 50 mm.

The felid partial skull with associated mandible discovered in the Late Villafranchian site of Monte Argentario (Tuscany, Italy) (Fig. 2) represents a key specimen for understanding this species’ functional morphology. Indeed, it shows a mosaic of intermediate characters between *Acinonyx* (e.g., the shortened snout) and *Panthera* (e.g., the massive canines). The latter features have led in the past to refer the skull to the leopard *Panthera pardus*^17^ and later to a female individual of the Pleistocene Eurasian jaguar *Panthera gombaszoegensis*^18^. However, the hard rock concretion in which the skull is embedded prevents a detailed analysis of its morphology. For this, it was necessary to resort to the most advanced technologies available in the field of tomographic investigations.

The recent diffusion of x-ray-based tomographic methods has opened new frontiers in palaeontology. For the first time, fossils can be virtually extracted from their host matrix and dissected, thus offering plenty of information on their external and internal morphology down to the micrometre scale. Obtaining 3D images can be easily achieved through computed tomography (CT) medical scanners, but these devices can provide limited spatial resolution (i.e. not smaller than 0.5 mm). Laboratory microtomography (µCT) has also been used in recent years for the study of fossils. However, despite the very good resolution of some of these machines, their polychromatic x-ray source is not suitable for analysing some fossils, such as highly mineralized samples with very low absorption contrast. The top technology available today for this kind of study is the scanning of fossils with synchrotron radiation (SR). The physical properties of the hard x-rays used for synchrotron microtomography (SR-µCT) (i.e., monochromaticity, high beam intensity and partial coherence) allow increasing significantly the data quality and imaging possibility compared to any other x-ray-based scanner^19^.

Here, we report on the results of the microtomographic analysis of the felid skull from Monte Argentario based on SR technology (applied for the first time on a Plio-Pleistocene carnivoran) and discuss about the morphological variation and ecology of *A*. *pardinensis*.

## Results

### External observations

The specimen (ArgBsc1) is represented by the rostral portion of the skull with associated mandible of a large felid (Fig. 2, Table 1). The neurocranium is almost completely missing, with only a small portion of the left frontal bone being visible. The splanchnocranium is better preserved on the left side, from which it looks rather shortened and dorsoventrally expanded. The zygomatic arches are lacking, as well as the orbits, with the exception of the dorsal margin of the left one, which is delimited by the postorbital process of the frontal. In rostral view, the nasal apertures look broad though slightly distorted, with the median part of the nasal bones being crushed into the nasal cavities. The mandible preserves the complete right corpus articulated with the anterior portion of the left one, broken at the level of the distal margin of the p4. Two mental foramina are visible on both sides, located at the same height immediately behind the distal margin of the lower canine and a few centimetres behind, respectively. Both the ascending rami are lost.

The teeth are virtually unworn and in variable state of conservation. Only the roots of the right I3 and i3 are visible in rostral view. The left canines are missing, but the distoventral portion of the lower canine root is still preserved, delimiting a wide pulp cavity. Conversely, the canines on the right side are almost intact and stand out to be robust and elongated. A short distance separates the upper canine from the P3. The latter is broken on the right side, but complete on the left. It is characterised by a high, vertical, and pointed paracone. The right P4 is a large and robust tooth. In labial view, it shows, from front to back, a strong parastyle, a high and vertically-oriented paracone and a slightly shorter metacone. The root of the small M1 is visible distally to the right carnassial. With the exception of the canine, the other lower teeth are impossible to describe because they are covered by the upper teeth or the embedding matrix. The latter is a highly consolidated red-rust coloured claystone very hard to remove mechanically without the risk of damaging the fossil. This led to the need to examine the specimen through a highly performing x-ray image analysis technique.

### Observations with SR-µCT

**Figure 3:**
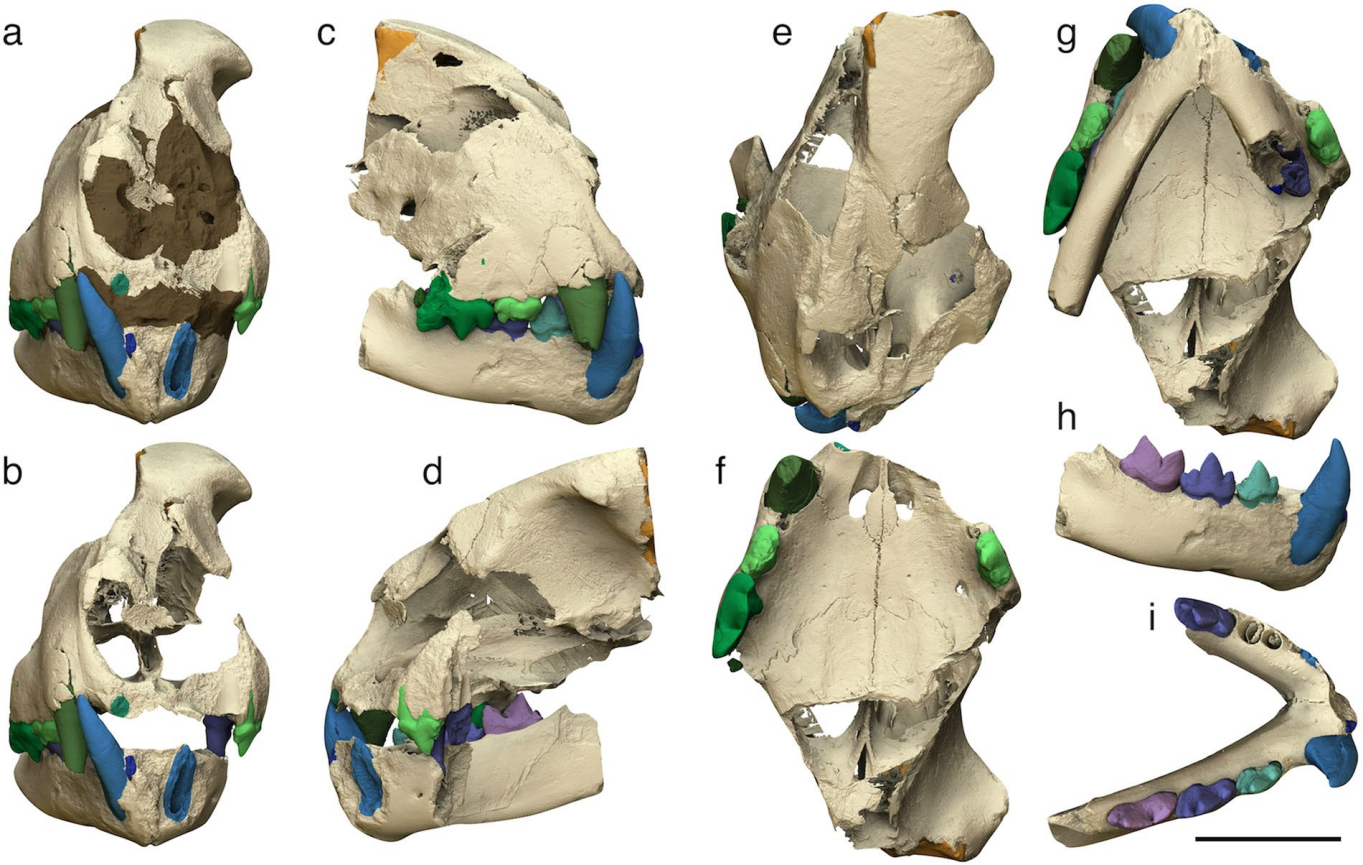
3D models of the *Acinonyx pardinensis* skull from Monte Argentario. (**a**,**b**) Rostral view of the skull with (**a**) and without (**b**) the embedding sediment (coloured in brown); (**c–e**) Skull in right lateral (**c**), left lateral (**d**), and dorsal (**e**) views; (**f**–**g**) Cranium in ventral view without (**f**) and with (**g**) the mandible; (**h**–**i**) Mandible in labial (**h**) and occlusal (**i**) views. Scale bar: 50 mm.

The scanning of the felid skull through SR at the European Synchrotron Radiation Facility (see ‘Methods’) allowed obtaining extremely clear and detailed images of the outer and inner structure of the specimen. By processing these images, we reconstructed a high-resolution 3D model of the skull, from which the matrix (coloured in brown in Fig. 3) can be digitally removed thanks to the segmentation process (see ‘Methods’).

**Figure 4:**
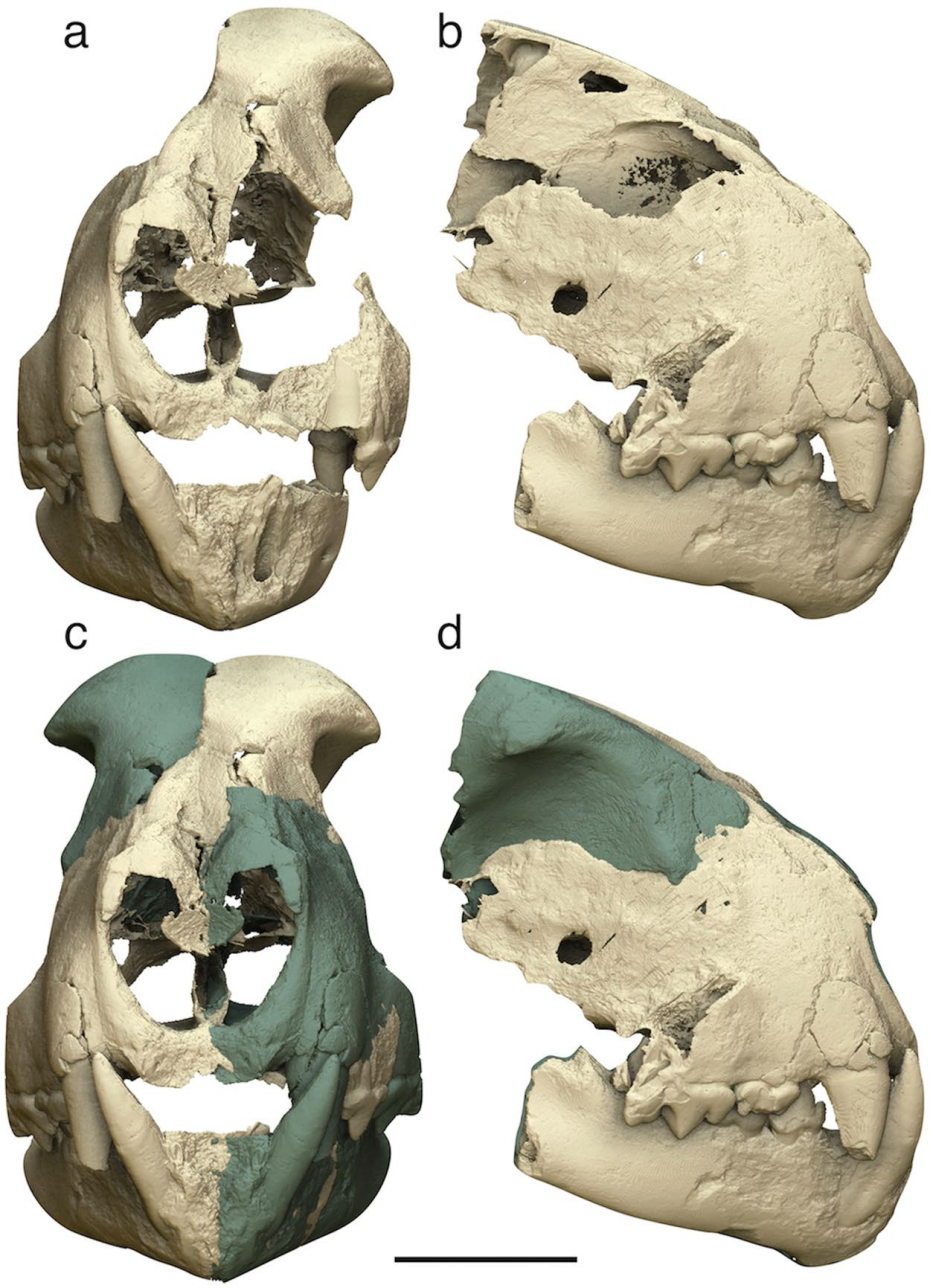
3D models of the *Acinonyx pardinensis* skull from Monte Argentario. (**a**,**b**) Damaged and fragmented skull in rostral (**a**) and right lateral (**b**) views: (**c**–**d**) Reconstructed skull with cloned and mirrored portions highlighted in different colour, in rostral (**c**) and right lateral (**d**) views. Scale bar: 50 mm.

The virtual model reveals many additional morphological details. Although the dorsal part of the skull is damaged, it was possible to three-dimensionally reconstruct the anterior portion of the left frontal sinus (coloured in yellow in Fig. 3). In ventral view, after removing virtually the mandible, the palate appears flat and wide. Rostrally, two relatively broad and elongated palatine fissures are visible. Palatal sutures are very well exposed. The suture between the palatal processes of the premaxilla and maxilla is divided into two segments by the palatine fissure. The lateral segment runs from the mid lingual margin of the canine alveolus to the caudolateral margin of the palatine fissure, describing a right angle; the medial segment runs steep towards the median palatine suture and forms a V shape with the homologous segment on the opposite side. The transverse palatine suture has a characteristic irregular pattern, with an indentation towards the medial side in the caudolateral part (in correspondence of the distal portion of P4) and another caudally-oriented indentation in the rostral part (which describes a W shape with the suture segment on the opposite side). The virtually reconstructed skull in Fig. 4 was obtained through the mirroring of the best-preserved portions on the opposite sides (i.e., the left orbital/frontal area was mirrored on the right side and the right premaxilla, maxilla, nasal fragments, and mandible were mirrored on the left side). Observing this model in rostral view, it can be noticed that (1) the nasal openings are actually narrower than they could appear on the original fossil, (2) the upper canines are housed in prominent canine eminences, and (3) the frontal region between the postorbital processes is rather narrow. The mandible is shortened and slender on the whole. In occlusal view, the symphysis is short and the corpi form a relatively wide angle. The right corpus is straight both in occlusal and labial view. From the latter perspective, the rostroventral margin of the mandible appears squared.

**Figure 5:**
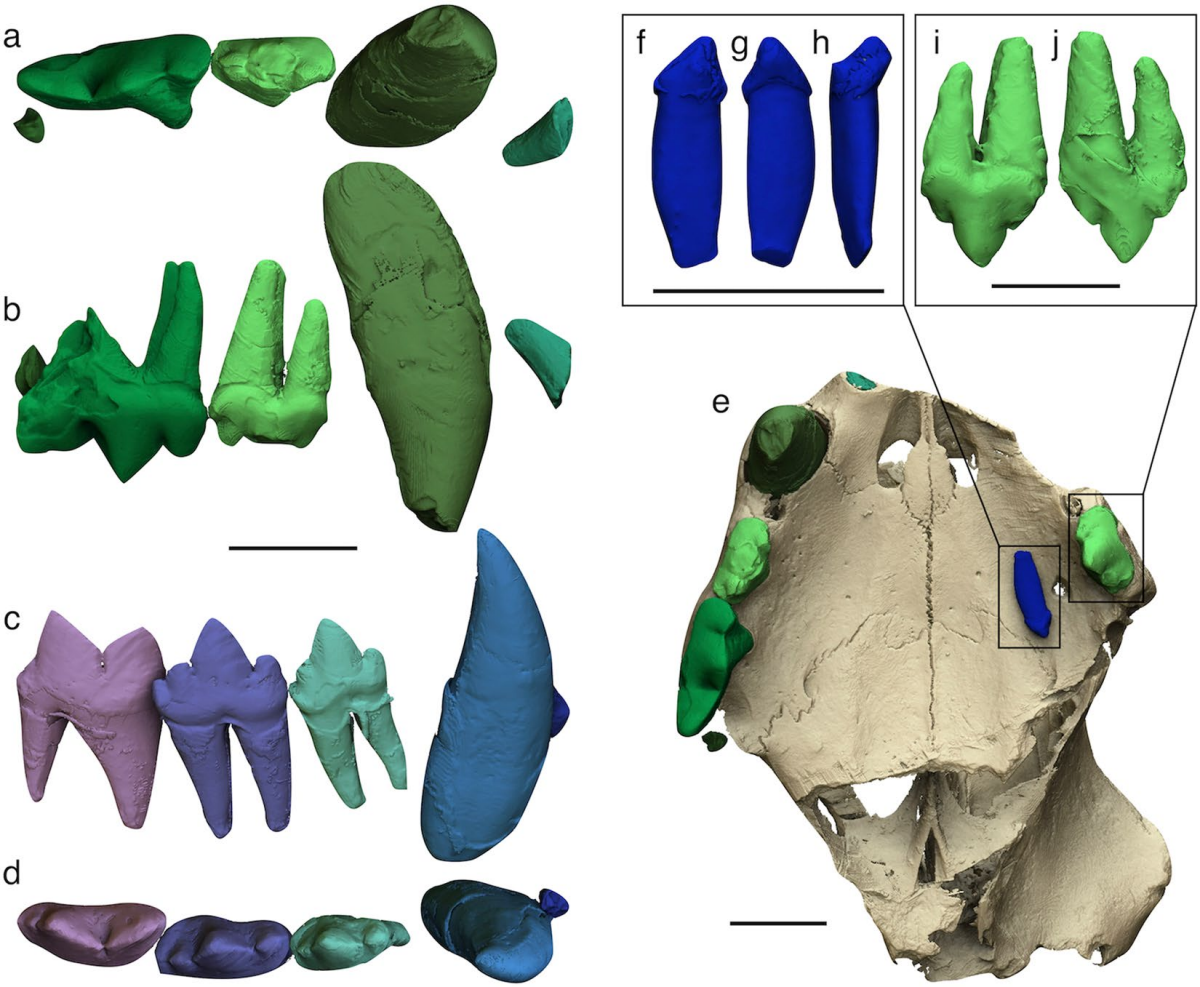
3D models of the teeth of *Acinonyx pardinensis* from Monte Argentario. (**a**,**b**) Right upper teeth in occlusal (**a**) and labial (**b**) views (the fragmented roots of I3 and M1 are preserved); (**c**,**d**) Right lower teeth in labial (**c**) and occlusal (**d**) views (the fragmented root of i3 is preserved); (**e**) Ventral view of the cranium showing the position of the left i3 lying against the left lateral surface of the palate; (**f**–**h**) Isolated left i3 in labial (**f**), lingual (**g**), and distal (**h**) views; (**i**,**j**) Left P3 in labial (**i**) and lingual (**j**) views. Scale bars: 30 mm.

The processing of SR-µCT images allowed to obtain single 3D models of all the teeth (Fig. 5) and separate them from the rest of the skull to facilitate the description. In occlusal view, the upper canine appears long and stout. The P3 is quite narrow and shows a small distolingual protocone. In the distal part of the tooth, a well-developed distal accessory cusp is aligned with the large and vertical paracone and the cusp-like distal cingulum is well defined. A small mesial accessory cusp is also visible on the left P3. The P4 shows a strongly reduced protocone, which has the appearance of a simple mesiolingual swelling of the crown. Two small circular alveoli reveal the previous presence of the P2 on both sides, which was strictly included between the upper canine and the P3. As for the lower teeth, the crowns of p3, p4, and m1 have almost the same height in labial view. The premolars show a ‘fleur-de-lis’ morphology, since the paraconid and the hypoconid diverge mesially and distally, respectively, from the dominant lanceolate protoconid. Both the p3 and p4 show a distinct distal cingulum. The length of the lower postcanine teeth increases gently from p3 to m1. In the latter, the sharp and triangular protoconid and paraconid have almost the same length. Distal to the protoconid, there is a well-defined talonid bearing a small tubercle in line with the main blade. In occlusal view, they are very close to each other so as to be partially overlapped. the distal portion of both premolars is wider than the mesial one.

**Figure 6:**
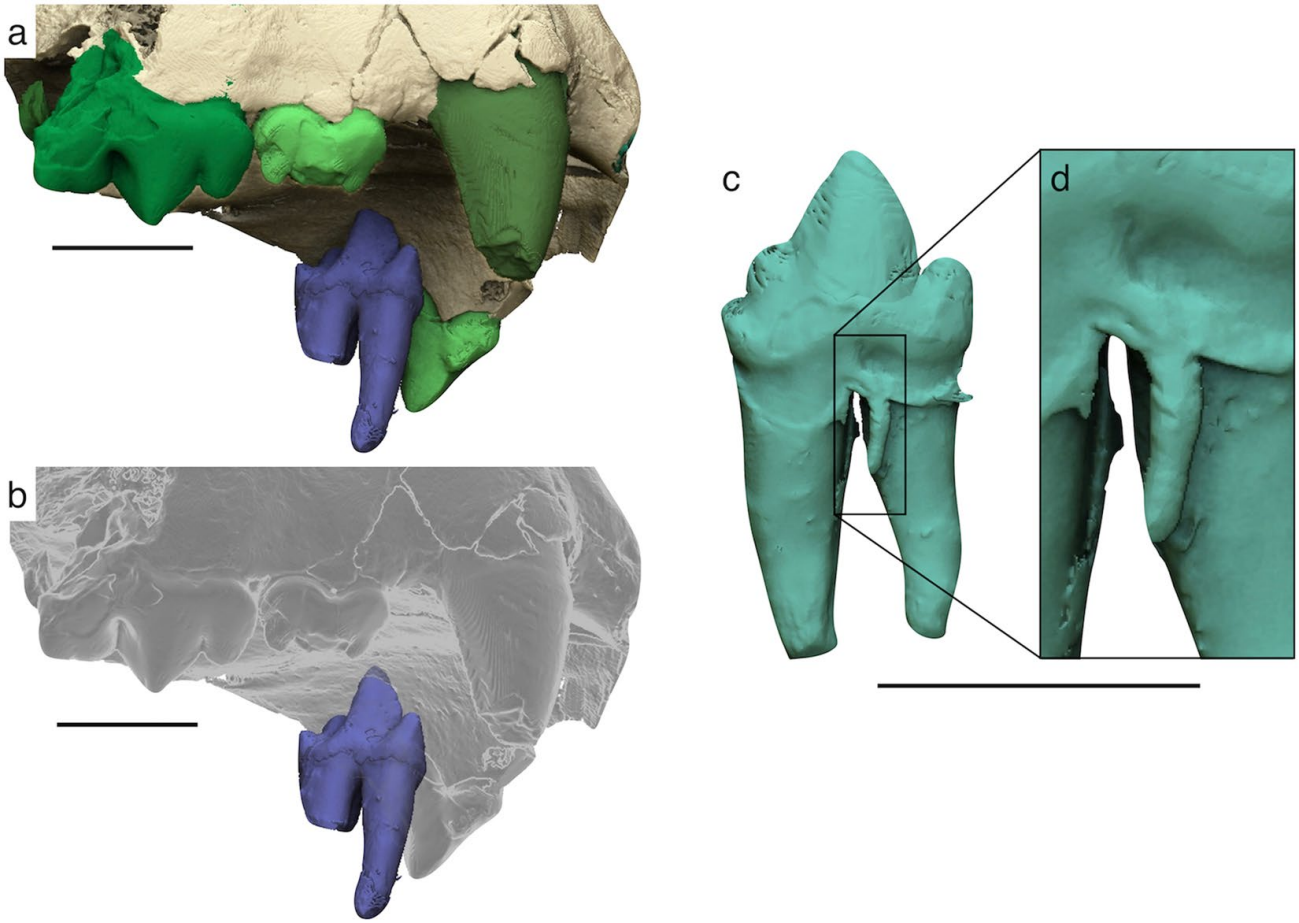
3D models of the teeth of *Acinonyx pardinensis* from Monte Argentario. (**a**,**b**) Detail of the left p4 piercing the palatine process of the left maxilla (semi-transparent cranium in **b**); (**c**) Small supernumerary and non-functional root developing on the labial side of the right p3; (**d**) Detail of the supernumerary root. Scale bars: 30 mm.

The analysis of µCT images evidenced the presence of an isolated tooth lying against the left lateral surface of the palate (Fig. 5e–h). The tooth is the left i3, which apparently slipped out of its alveolus before the final lithification of the enclosing sediments. Similarly, some kind of pre-diagenetic stress must have pushed towards the cranium the left p4, which came out partially from the alveolus and pierced the palatine process of the left maxilla with its pointed protoconid (Fig. 6a–b). A tiny supernumerary root develops on the labial side of the right p3 where the two main roots join the crown (Fig. 6c–d). Its reduced size and the absence of the pulp cavity indicate that it is a non-functional root. Such a condition has been reported in some extant and fossil carnivorans^20–23^, although the origin of this anomaly is not clear because patterning mechanism of the root position and number is still poorly known^24^.

## Discussion

The identification of the specimen (ArgBsc1) based solely on external observation is hard, since the enclosing in the rock matrix makes very difficult the analysis of diagnostic characters, especially in the teeth. From the outside, the skull shows a series of intermediate *Acinonyx*-like and *Panthera*-like characters. The first include the high and short muzzle, the straight ventral margin and the right rostroventral angle of the mandible. The second include the relatively narrow frontals and especially the massive canines, mainly the upper ones, housed in prominent canine eminences. In particular, the latter characters might have led to the past erroneous attribution of the specimen to *P*. *gombaszoegensis*^18^, whose stratigraphic occurrence partially overlaps that of *A*. *pardinensis*. This may have been due also to the limited past knowledge of this taxon’s anatomy, which has increased significantly thanks to new discoveries in recent decades. In this regard, it is worth mentioning two other cases of misleading identification involving the same taxa. The first concerns Pietrafitta, a central Italian site with an age comparable to that of Monte Argentario^25^, the second the slightly older locality of Ellera di Corciano^26^. The scarce felid material from the two sites were referred in the past to *P*. *gombaszoegensis*^27–30^ and cf. *P*. *gombaszoegensis*^31,32^, respectively. The fossils are here reassigned to *A*. *pardinensis* (Supplementary Note 1 and Supplementary Figs S1–S2).

The study of images obtained through the SR-µCT survey resulted in many additional information for the definition of the taxonomic status of the Monte Argentario skull. The following craniodental features identified on the 3D models of ArgBsc1 are consistently found in the other specimens attributed to *A*. *pardinensis*^6,12,33^ and differ from pantherine felids:

- Short and wide palate;
- Relatively short and slender mandible, with reduced diastema between the lower canine and the p3 and short symphysis;
- Crowded and partially overlapped upper and lower postcanine teeth;
- Narrow P3 with sub-parallel labial and lingual margins in occlusal view, and high, pointed, and sub-vertical paracone;
- P4 characterised by the strong reduction of the protocone, which is only slightly protruding lingually and appears like a prolongation of the lingual root toward the crown base;
- Cuspids of p3 and p4 exhibiting a ‘fleur-de-lis’ morphology;
- Lower postcanine teeth with similar crown height in labial view.

Similarly, most of the above characters allow to exclude that the Monte Argentario specimen can be referred to the late Pliocene-Early Pleistocene puma-like felid *Puma pardoides*^34,35^. In fact, although the latter species shows some similarities with *A*. *pardinensis* (e.g., lateral enlargement of the frontals caudally to the zygomatic processes and relatively shortened muzzle)^6,33^, differences between them are remarkable (e.g., *Pu*. *pardoides* has labiolingually enlarged P3, P4 with strong *Panthera*-like protocone, p3 significantly lower than p4 in labial view both showing more massive protoconid)^5,6^. Moreover, the overall size of *Pu*. *pardoides* is significantly smaller than that of *A*. *pardinensis* (the upper carnassial of *Pu*. *pardoides* is about 22 mm in average length^5^, while that of *A*. *pardinensis* is about 30 mm^6^).

In the light of the above features, the felid from Monte Argentario is here referred to *A*. *pardinensis*.

However, the occurrence of *Panthera*-like characters in the skull is noteworthy. Among them, the presence of stout and strong canines stands out. The linear dimensions (length and breadth) of these teeth fall in the uppermost part of the *A*. *pardinensis* range and, at least for the upper canine, are close to the lower values recorded for medium-sized pantherines like *P*. *gombaszoegensis* (Supplementary Tables S1–S2 and Supplementary Fig. S3). Similarly, the comparative analysis of the palatal sutures (clearly visible in ArgBsc1 for the first time in *A*. *pardinensis*) evidences further affinities with pantherine cats. In particular, the indented suture trend observed in the Monte Argentario skull recalls more the extant leopard and snow leopard than the cheetah and puma (Supplementary Fig. S4).

Previous studies^6,10,12^ already highlighted that *A*. *pardinensis* retains *Panthera*-like, primitive morphological characters, but most of them relate to the neurocranium (e.g., the relatively elongated braincase, with high sagittal and nuchal crests and—as also visible in ArgBsc1—narrow frontals between the postorbital processes). The specimen from Monte Argentario shows some significant pantherine-like characters also in the splanchnocranium (i.e., teeth and palatal sutures). Nevertheless, at least as regards to canine dimensions, our results should be placed in the context of the overall *A*. *pardinensis* intraspecific variation. As in part predictable by the long-lasting stratigraphic distribution (from late Pliocene to Middle Pleistocene) and the wide geographical distribution (from Morocco to China), *A*. *pardinensis* exhibits a considerable variation in size, with body mass estimated (for adult individuals) from about 60 to 120 kg (Supplementary Table S3 and Supplementary Fig. S4). This variation led some scholars^15,33,36^ to consider *A*. *pardinensis* as a broad ‘macrospecies’ subdivided into several subspecies^6^: *A*. *p*. *arvernensis* from Les Étouaires (late Pliocene) and Tuozidong (early Early Pleistocene); *A*. *p*. *aicha* from North Africa (earliest Pleistocene); *A*. *p*. *pardinensis* from a number of European to central Asian sites (Early Pleistocene; Middle-Late Villafranchian LMA); *A*. *p*. *linxiaensis* (=*‘Sivapanthera’ linxiaensis*)^10^ from northern China (early Early Pleistocene); *A*. *p*. *pleistocaenicus* from Untermassfeld (late Early Pleistocene; Epivillafranchian stage)^37^ and China (=*‘Sivapanthera’ pleistocaenica*)^11^ (Early Pleistocene; Villafranchian LMA); *A*. *p*. *intermedius* from Hundsheim and Mosbach (Middle Pleistocene; Galerian LMA). However, the validity of this taxonomic model is questioned by the lack of clear morphological or biometrical differences between the different subspecies. The available estimated body masses for *A*. *pardinensis* suggest that the variation found is independent of the age and the geographical location of the finds (Supplementary Fig. S5). An emblematic case is represented by the earliest (i.e., Early Villafranchian LMA) European specimens attributed to this taxon, namely those of Villafranca d’Asti (Supplementary Fig. S6), Villarroya, and Les Étouaires, for which the predicted body masses are 70, 87, and 121 kg, respectively. Unfortunately, the existing data are not sufficient to understand if the intraspecific variation of *A*. *pardinensis* could be linked to ecogeographical dynamics and/or sexual dimorphism. However, actualistic comparisons with extant large-sized felids allow to hypothesise that the latter factor can be called into question for *A*. *pardinensis*. Sexual dimorphism in body mass occurs in all living felids, and is particularly marked in wide-range large-sized species. For example, the average weight difference between males and females is about 60% in the jaguar, 80% in the leopard, 85% in the lion and the tiger, and even 90% in the puma, while it is much smaller (~15%) in the cheetah^38^. We obtain interesting information if we try to free the interpretations on *A*. *pardinensis* from the stratigraphic influence, that is if we consider the few available sites for which it is possible to estimate body mass for more than one individual (which we can therefore hypothesise as being coeval). The difference between the lowest and highest predicted body mass is about 31% at Ahl al Oughlam, 33% at Saint Vallier, and 72% at Longdan. This suggests that sexual dimorphism may have played an important role in the intraspecific variation of *A*. *pardinensis*, as hypothesised by Petrucci *et al*.^39^ based on postcranial material from Pirro Nord. Although out of the scope of this work, the same can be said for the Eurasian jaguar *P*. *gombaszoegensis*, whose predicted body masses span from about 65 to 180 kg in all its chronological (about 1.9–0.3 Ma) and geographic (Europe and Western Asia) range, with peaks of variation of ~80% and even 90% for local samples such as those from the type locality of Gombaszög and Untermassfeld, respectively (Supplementary Table S4).

*Acinonyx pardinensis* and *P*. *gombaszoegensis* co-occurred in Europe for about 1.4 Ma in the Early and Middle Pleistocene, and are reported together in at least seven sites (Olivola, Dmanisi, Upper Valdarno, Pirro Nord, Untermassfeld, Le Vallonnet, and Mosbach, in decreasing stratigraphical order), often in association with sabretooth cats like *Megantereon* and/or *Homotherium*^6,39,40^. This extraordinary concentration of large felids is unequalled in modern ecosystems and, moreover, must be included in the context of the rich diversity of the Late Villafranchian/Epivillafranchian large carnivore guilds, which included, among others, also hyaenids and canids^41^. Interspecific competition for prey must have been strong in such palaeoecosystems and subject to ecological mitigation strategies. These could have included temporal niche displacement (e.g., diurnal/nocturnal hunting) and/or different spatial distribution within the ecosystem^6^ (for example, in Venta Micena, geochemical data suggest that *Megantereon* and *Panthera* preferred forested areas whereas *Homotherium* and *Lycaon* hunted in more open habitats)^42^. The recognition of pantherine-like characters in the Monte Argentario felid—as well as in other *A*. *pardinensis* samples from other localities—must lead to reconsidering the ecological role of this species, whose choice of prey and hunting strategy was likely to be different from those of the living cheetah^6,16^. Furthermore, we hope that the approach followed in this work, i.e., the use of high-resolution µCT techniques, may also be followed in the future for similar cases, in order to extract as much information as possible from fossil remains of Plio-Pleistocene vertebrates.

## Methods

### Material

The investigated specimen of *A*. *pardinensis* (ArgBsc1) is housed in the PaleoFactory, Sapienza University of Rome (Italy). The analytical study of the fossil has been carried out in 2015 in agreement with the former Archaeological Superintendence of Tuscany (Italy).

### Locality

Monte Argentario is a small promontory on the Tyrrhenian coast of southern Tuscany (Italy). The outcropping Permian-Triassic limestones and dolomites^43^ were subjected by karst processes in recent geological times. Karst cavities and fissures are filled by strongly cemented conglomerates and red clay-sand layers^25^. In the locality of “Miniera della Polveriera”^17^, the latter layers yielded a diversified assemblage of terrestrial vertebrates referred to the Late Villafranchian LMA (Farneta-Pirro Faunal Units; ~1.5 Ma)^25,44^. The assemblage includes the following taxa: Large mammals^18,44–46^—*Ursus etruscus*, *Canis* sp., *Pachycrocuta brevirostris*, *Lynx issiodorensis*, *Acinonyx pardinensis*, *Homotherium* sp., *Megantereon whitei*, *Stephanorhinus* cf. *S*. *hundsheimensis*, *Pseudodama* sp., *Leptobos* sp., *Soergelia* cf. *S*. *minor*, Bovidae indet. Small mammals^25^—*Talpa* cf. *T*. *fossilis*, *Sorex* cf. *S*. *minutus*, *Petenyia hungarica*, *Asoriculus gibberodon*, *Oryctolagus* cf. *O*. *valdarnensis*, *Eliomys* cf. *E*. *intermedius*, *Glis sackdillingensis*, *Microtus* (*Allophaiomys*) cf. *M*. (*A*.) *ruffoi*, *Victoriamys chalinei*, *Apodemus* (*Sylvaemus*) *sylvaticus*, *Apodemus* (*S*.) *flavicollis*, *Apodemus* (*S*.) gr. *sylvaticus-flavicollis*, and *Apodemus* (*Karstomys*) gr. *mystacinus-epimelas*. Birds^25^—*Anas* sp., *Alectoris* cf. *A*. *graeca*, *Porzana parva* vel *P*. *pusilla*, *Columba livia* vel *C*. *oenas*, *Bubo* sp., Passeriformes indet. Reptiles^25^—*Lacerta* sp. Lacertilia indet., “Colubrines” indet., *Vipera* sp., Serpentes indet. Amphibians^25^—cf. *Bufo bufo*.

### Imaging

The skull was characterised using propagation phase contrast synchrotron microtomography at the ID17 beamline of the European Synchrotron Radiation Facility (ESRF, Grenoble, France; proposal HG37). The setup consisted of a monochromatic beam (double bent Laue) at 135 keV using a Wiggler 150 (gap 24.9 mm) in 16 bunch mode, 2.56 mm of vitreous carbon and 5.59 mm of Cu to reduce the heat load on the monochromator, 10 m of propagation between the sample and the detector, and an optical system producing data with a pixel size of 46.19 µm (scintillating optical fibre, ~0.26x magnification set of lenses, FReLoN-2k in frame transfer mode). As the skull did not fit in the horizontal field of view (FOV), the rotation axis was shifted from the centre of the image toward the right edge of the FOV by ~37 mm (i.e., half-acquisition protocol). The tomographic acquisition consisted of 8000 projections (to accommodate the increase of the reconstructed slices in half-acquisition) of 0.2 seconds each over 360°, 101 projections for flat field images and 100 images for the dark noise correction. Given the limited vertical FOV (5.54 mm) several scans were needed, moving the specimens along the vertical axis of the sample stage in between each scan. In order to correct for the vertical profile of the x-ray beam and provide useful data for ring artefact correction, the samples were moved each time by 2.5 mm, hence offering an overlap of 50% of two consecutive scans. In this configuration, 75 scans were necessary to scan the specimen. During the vertical stitching of the scans, done on the reconstructed slices, the overlapping part was handled by a weighted average, giving more weight to slices reconstructed from the centre of the projections. The tomographic reconstruction was done using PyHST2^47^, first as a stack of 32-bit data. Then the dynamic range was changed to 16 bits using the min and max 0.2% saturation values from the 3D histogram calculated by PyHST2. The 16-bit stacks of tiff were stitched together and finally a ring correction was applied^48^.

2,009 slices were obtained, which were downsampled by half and segmented using the software Materialise Mimics (version 17.0). The 3D rendering of the model of ArcBsc1 and the partial reconstruction of the missing portions of the skull were performed using the software ZBrush (version 4R6). All the missing parts of the specimen were cloned and mirrored from those preserved, and their alignment and positioning were performed following the sagittal plane of the skull. Finally, the reconstructed portions were highlighted using a different colour.

**Table 1:** Craniodental measurements (mm) of *Acinonyx pardinensis* from Monte Argentario. R, right; L, left. Values in italics are estimated.

Cranium and upper teeth			Mandible and lower teeth		
Frontal breadth	45		Height behind the canine	33.2	
Min breadth between the orbits	43		Height behind m1	33.1	
Breadth at the canine alveoli	77		Breadth at p4	14.9	
Max palatal breadth (at the P4 alveoli)	100		Length of the diastema	7.7	
Alveolar length of the cheektooth row	55		Alveolar length of the cheektooth row	59.6	
Alveolar length of the premolar row	53			**R**	**L**
	**R**	**L**	i3 length	—	5.1
C length	17.8	—	i3 breadth	—	4.5
C breadth	13.2	—	c length	14.9	—
P3 length	17.8	16.9	c breadth	12.5	—
P3 max breadth	7.9	7.9	c crown height	27.9	—
P3 mesial breadth	7.7	7.6	p3 length	15.1	—
P3 paracone length	—	7.6	p3 max breadth	8.7	—
P3 paracone height	—	12.6	p3 mesial breadth	6.4	—
M1 length	30.0	—	p3 protoconid length	8.3	—
M1 length at the protocone	25.3	—	p3 protoconid height	11.9	—
M1 max breadth	13.7	—	p4 length	20.3	*19*.*0*
M1 breadth behind to protocone	10.4	—	p4 max breadth	10.2	9.7
M1 max distal breadth	10.4	—	p4 mesial breadth	8.4	8.0
M1 paracone length	11.1	—	p4 protoconid length	8.7	8.2
M1 metacone length	11.6	—	p4 protoconid height	13.8	13.7
M1 paracone + metacone length	21.7	—	m1 length	22.6	—
M1 paracone height	16.0	—	m1 max breadth	9.4	—
			m1 protoconid length	10.6	—
			m1 protoconid height	14.1	—
			m1 paraconid length	8.7	—
			m1 paraconid height	14.1	—
			m1 height at the central notch	7.7	—

## Electronic supplementary material

Supplementary Information
