# Supplementary material for: Synchrotron radiation reveals the identity of the large felid from Monte Argentario (Early Pleistocene, Italy)
Source: Sci Rep. 2018 May 29;8:8338. doi: 10.1038/s41598-018-26698-6 (PMC5974229; doi:10.1038/s41598-018-26698-6)
Supplement: Supplementary file 1 — Supplementary Information [file 41598_2018_26698_MOESM1_ESM.pdf]

Supplementary Information

Synchrotron radiation reveals the identity of the large felid from Monte Argentario (Early Pleistocene, Italy)

Marco Cherin, Dawid A. Iurino, Marco Zanatta, Vincent Fernandez, Alessandro Paciaroni, Caterina Petrillo, Roberto Rettori & Raffaele Sardella

Supplementary Note 1: *Acinonyx pardinensis* from Pietrafitta and Ellera di Corciano

The Late Villafranchian vertebrate collection from Pietrafitta (Umbria, Italy) constitutes the richest and most diversified local fauna of the Farneta Faunal Unit (~1.5 Ma) in Italy, being formed by hundreds of remains of freshwater fishes (Cyprinidae), amphibians (*Latonia* cf. *L. regei* and *Rana* gr. *R. ridibunda*), reptiles (*Vipera* cf. *V. ammodytes*, *Natrix* sp., Colubrines indet., and *Emys orbicularis*), birds (*Podiceps* sp., *Phalacrocorax* sp., cf. *Ixobrychus*, *Cygnus* sp., *Anas* sp. (large size), *Anas crecca/querquedula*, *Aythya* sp., *Somateria* aff. *S. mollissima*, Rallidae indet., cf. *Gallus*), and especially mammals (*Sorex* cf. *S. minutus*, *Oryctolagus* cf. *O. lacosti*, *Mimomys pusillus*, *Microtus* (*Allophaiomys*) cf. *M.* (*A.*) ruffoi, M. (*A.*) *chalinei*, *Castor fiber plicidens*, *Macaca sylvanus florentina*, *Ursus etruscus*, *Panthera gombaszoegensis*, *Pannonictis nestii*, *Equus* sp., *Stephanorhinus* cf. *S. hundsheimensis*, *Leptobos* aff. *L. vallisarni*, *Pseudodama farnetensis*, *Praemegaceros obscurus*, and *Mammuthus meridionalis*)[29]. According to Gentili et al.[29], *P. gombaszoegensis* is represented at Pietrafitta by 'two fragments of carpals'. However, the only large felid specimen present to date in the collection is a non-numbered proximal fragment of left third metatarsal, whose morphology and size fit those of *A. pardinensis* from other European sites (Supplementary Fig. S1).

The poor mammal assemblage from "Filiale Lancia" at Ellera di Corciano (Umbria, Italy) is dubitatively referred to the Tasso Faunal Unit (~1.8 Ma) of the Late Villafranchian LMA. The assemblage includes *Stephanorhinus* cf. *S. etruscus*, *Hippopotamus* cf. *H. antiquus*, cf. *Eucladoceros* sp., *Pseudodama* sp., *Leptobos* cf. *L. vallisarni*, and cf. *P. gombaszoegensis*[31,32]. The latter taxon is represented by a complete left calcaneum (Supplementary Fig. S2). Calcanei of *P. gombaszoegensis* and *A. pardinensis* are rare in the fossil record. In her review of the first species, O'Regan[40] reports a left calcaneum of *P. gombaszoegensis* from Olivola (Italy) that is different from the Ellera specimen both morphologically and dimensionally (GL=73.1 mm, GB=30.1 mm; see Supplementary Fig. S2 for measurement explanation). According to the same author[40], other putative calcanei of *P. gombaszoegensis* from several European sites should be actually referred to other species. For instance, the morphology and size of the felid calcanei from Mosbach (Germany; GL=92+ mm, GB=30+ mm) and Petralona (Greece; GL=104 mm, GB=44 mm) suggest an attribution

to *A. pardinensis*. Similarly, the Uppony 1 (Hungary) and Château Breccia (France) calcanei might belong to the cave lion[40]. Also the recently published new felid material from the last site includes some calcanei referred to *P. gombaszoegensis*[1], but the length of the two most complete specimens (about 100 mm) exceeds that of medium-sized *Panthera* and suggests attribution to a larger species, such as *P. spelaea* (which co-occurs with *P. gombaszoegensis* at least in some parts of the Château deposit). We directly compared the specimen from Ellera di Corciano with the aforementioned calcaneum of *P. gombaszoegensis* from Olivola (IGF 854) and found several morphological differences: the medial articular facet for the astragalus is more rounded in IGF 854 and its vertical extension along the ventromedial margin of the bone is indistinct; the lateral articular facet for the astragalus is more expanded medially in IGF 854, so as to cover part of the lateral margin of the bone in anterior view; between the two articular facets, IGF 854 shows a deep depression. Conversely, the specimen from Ellera di Corciano fits the morphology and size of the calcanei of *A. pardinensis* from Untermassfeld (Germany) and especially Olivola (Supplementary Fig. S2). and is here reassigned to that species.

Supplementary Note 2: Institutional abbreviations

CCEC, Centre de Conservation et d'Étude des Collections, Lyon (France); **DFGP**, Dipartimento di Fisica e Geologia, Università di Perugia (Italy); **DGM**, Department of Geology, Universidad Complutense de Madrid (Spain); **DGT**, Department of Geology & Physical Geography, Aristotle University of Thessaloniki (Greece); **DGUA**, Department of Geology, University of Athens (Greece); **IVPP**, Institute of Vertebrate Paleontology and Paleoanthropology, Chinese Academy of Sciences (China); **HMV**, Hezheng Paleozoological Museum, China; **HNHM**, Hungarian Natural History Museum and Geological Institute of Hungary, Budapest (Hungary); **ICP**, Museo del Institut Català de Paleontologia 'Miquel Crusafont', Sabadell (Spain); **IGF**, Museo di Storia Naturale, Sezione di Geologia e Paleontologia, Università di Firenze (Italy); **IGME**, Instituto Geológico y Minero de España, Madrid (Spain); **INSAP**, Institut National des Sciences de l'Archéologie et du Patrimoine du Royaume du Maroc, Casablanca (Morocco); **IQW**, Senckenberg Research Station of Quaternary Palaeontology, Weimar (Germany); **LCOG**, Leakey Camp Osteological Collections, Olduvai Gorge (Tanzania); **LMN**, Landesmuseum Niederösterreich, St. Pölten (Austria); **MMSH**, Maison Méditerranéenne des Sciences de l'Homme,Aix-en-Provence (France); **MNCN**, Museo de Ciencias Naturales, Madrid (Spain); **MNHN**, Muséum National d'Histoire Naturelle, Paris (France); **MZUF**, Museo di Storia Naturale, Sezione di Zoologia 'La Specola', Università di Firenze (Italy); **NHM**, Natural History Museum, London (UK); **NHMB**, Natural History Museum Basel (Switzerland); **NML**, Naturalis Museum, Leiden (Netherlands); **NMM**, Naturhistorisches Museum Mainz (Germany); **NMNHS**, National Museum of Natural History, Sofia (Bulgary); **PF**, PaleoFactory, Sapienza University of Rome (Italy); **SBAU**,

Soprintendenza per i Beni Archeologici dell'Umbria, Perugia (Italy); **SMF**, Senckenberg Naturmuseum Frankfurt (Germany).

Supplementary figures

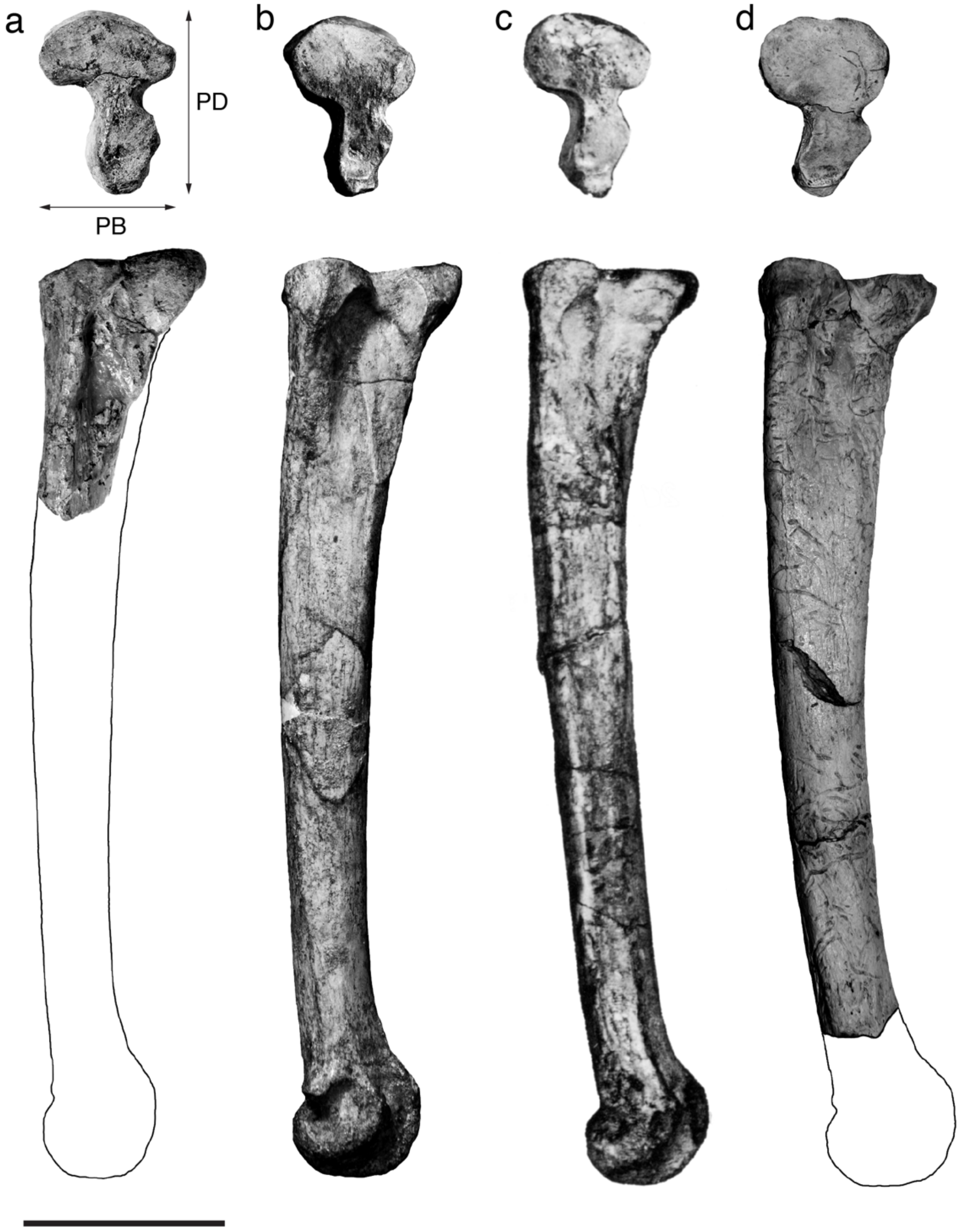

Supplementary Fig. S1. Third metatarsal of *Acinonyx pardinensis* from various European localities in proximal (above) and lateral (below) views. PB, proximal breadth; PD, proximal depth. (**a**) SBAU no num., Pietrafitta (Italy), PB=19.8 mm PD=25.3; (**b**) NHMB Prr191, Les Étouaires (France), PB=20.0 mm, PD=25.7 mm; (**c**) IGF 2613, Olivola (Italy), PB≈24 mm, PD≈26 mm (pictures modified from Ficcarelli[13]); (**d**) IQW 1980/15796 (Mei. 15167), Untermassfeld (Germany), PB=19.0 mm, PD=24.0 mm (measurements from Hemmer[14]). (**a**) and (**b**) are left; (**c**) and (**d**) are right but are figured reversed. Scale bar: 30 mm.

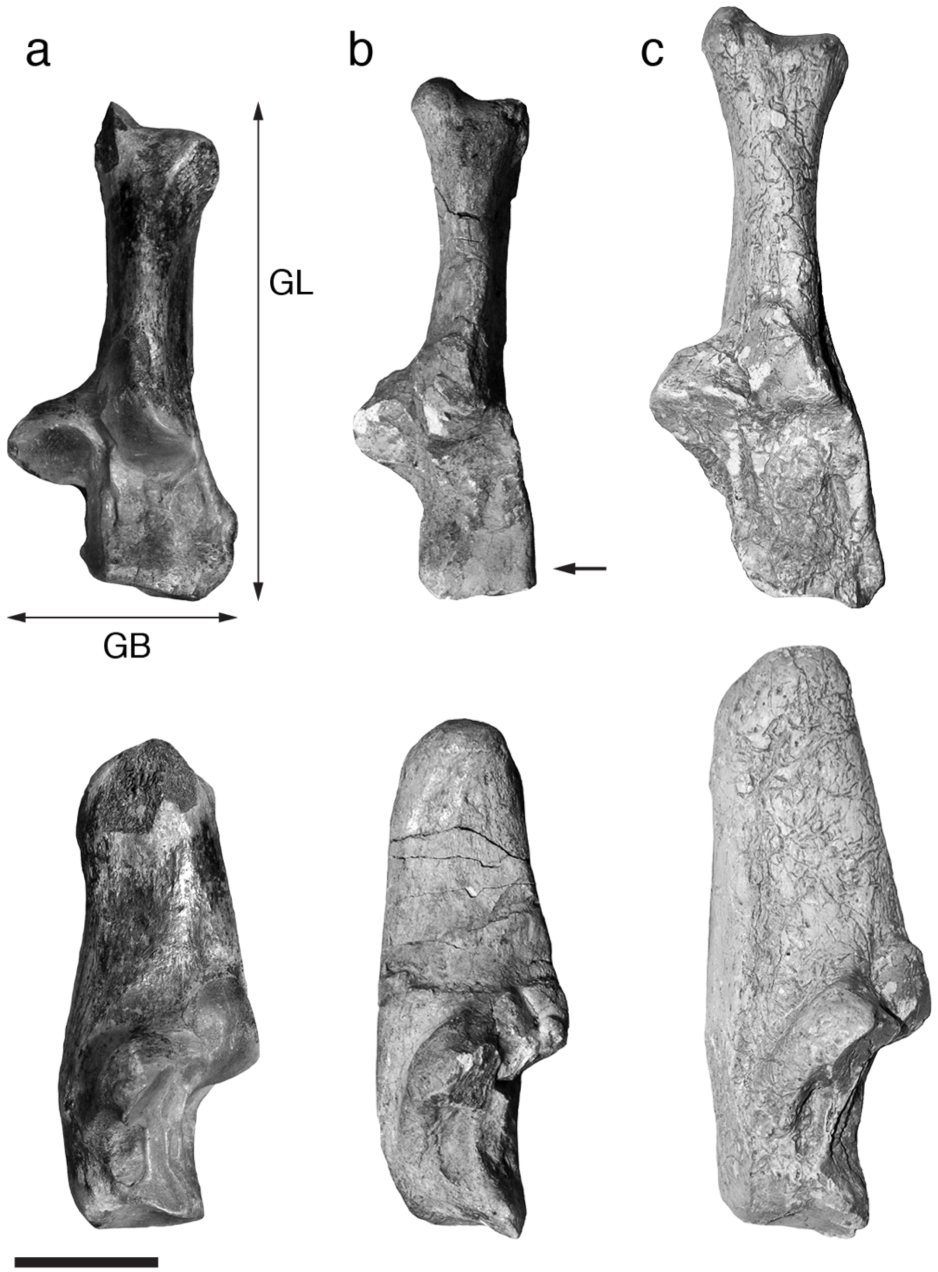

Supplementary Fig. S2. Calcaneum of *Acinonyx pardinensis* from various European localities in anterior (above) and medial (below) views. GL, greatest length; GB, greatest breadth. (**a**) SBAU no num., Ellera di Corciano (Italy), GL=95 mm, GB=44 mm; (**b**) IGF 2613, Olivola (Italy), GL=99 mm, GB=35+ mm (measurements from Ficcarelli[13]; the ventrolateral portion of the bone indicated by the black arrow, appears less developed than in the Ellera specimen but this might be due to the fragmentation of the that portion); (**c**) IQW 1980/15793 (Mei. 15305), Untermassfeld (Germany), GL=113 mm, GB=42 mm. (**a**) is left; (**b**) and (**c**) are right but are figured reversed. Scale bar: 30 mm.

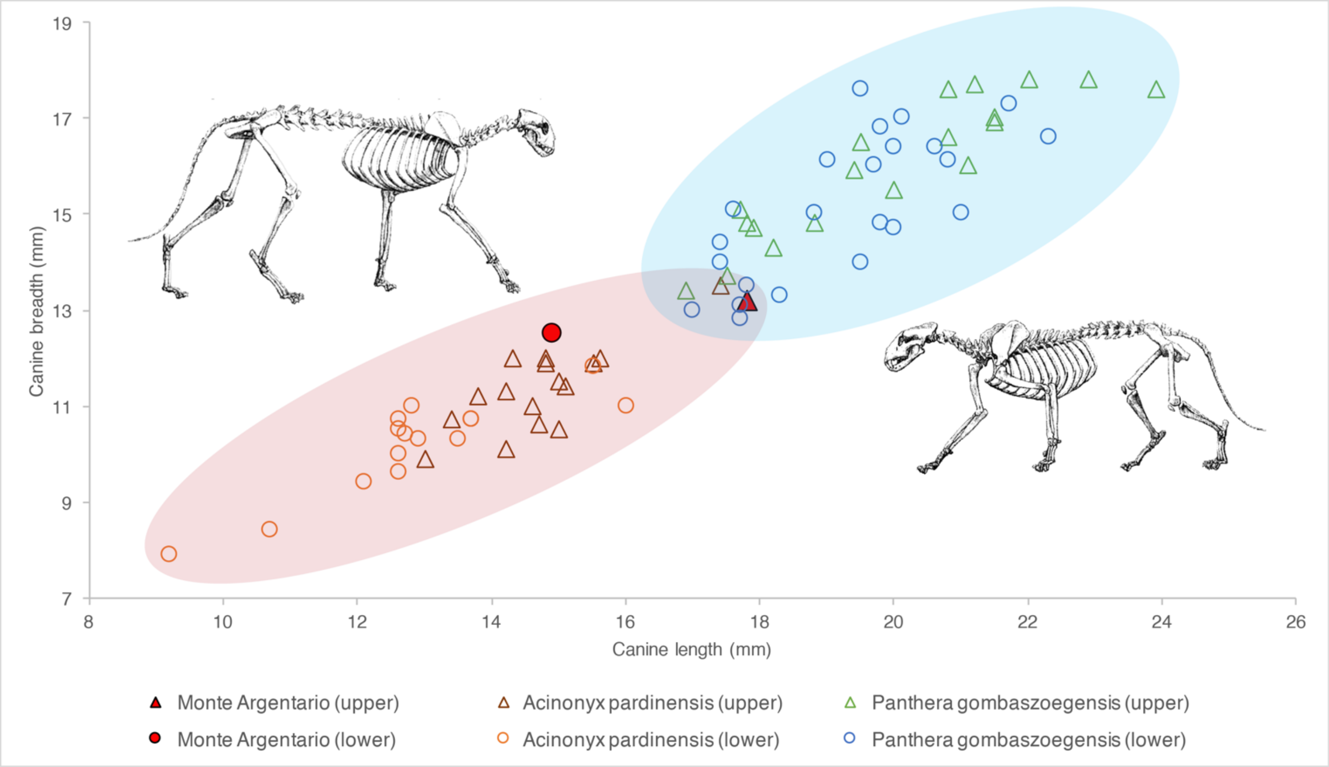

Supplementary Fig. S3. Scatter plot showing the relationship between length and breadth of the upper (△) and lower (○) canines in *Acinonyx pardinensis* (red area), *Panthera gombaszoegensis* (blue area), and Monte Argentario felid (full red symbols). Morphometric data used to build the chart are in Supplementary Tables S1 and S2. Skeletal reconstructions courtesy of Mauricio Antón.

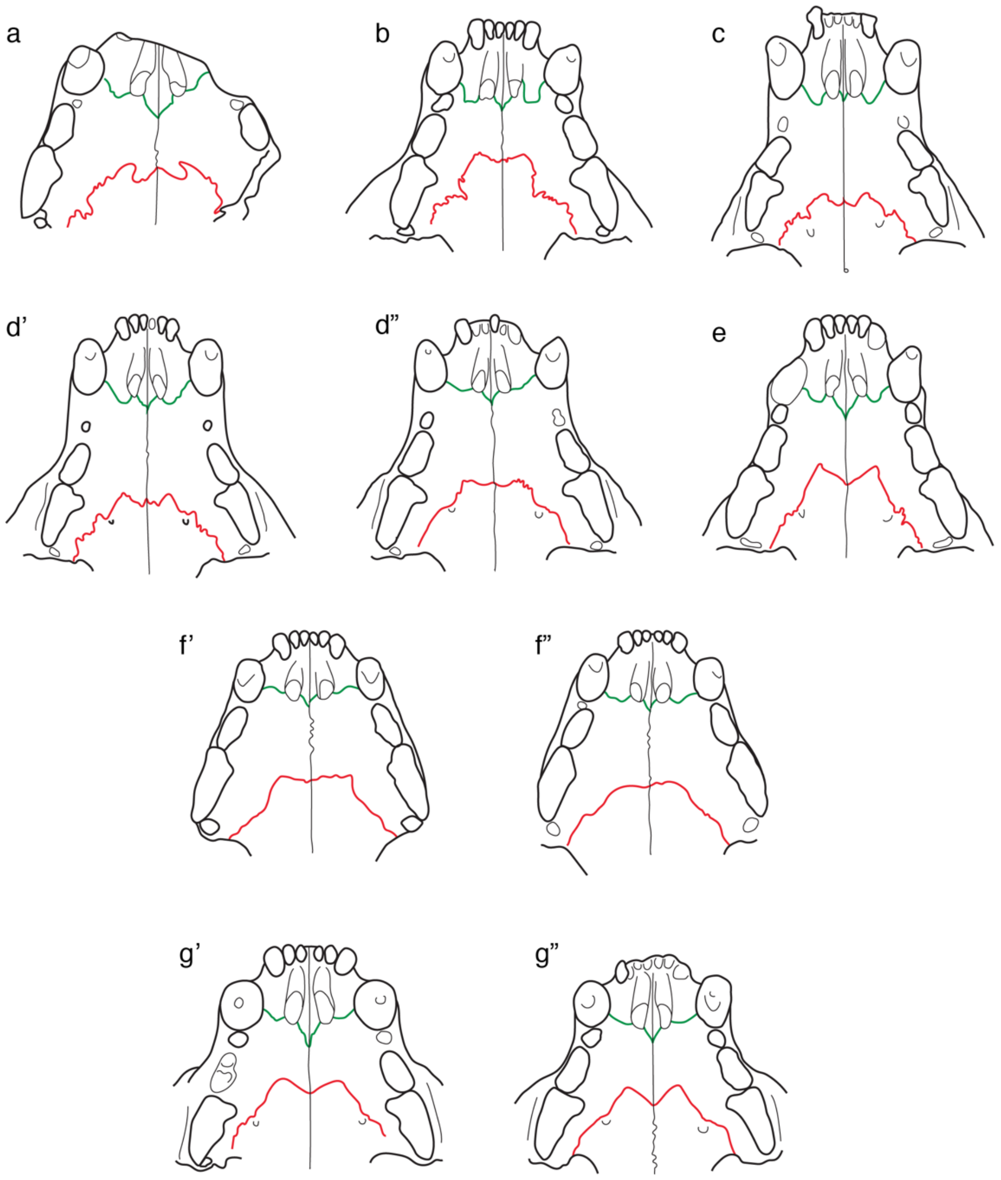

Supplementary Fig. S4. Ventral view of the palate of the Monte Argentario felid (**a**) and living medium to large-sized felids: (**b**) *Panthera onca* MZUF 501; (**c**) *Panthera uncia* NHM 75.2283; (**d’**) *Panthera pardus* HNHM 60.187.1; (**d”**) *Panthera pardus* LCOG no num.; (**e**) *Panthera leo* LCOG no num.; (**f’**) *Acinonyx jubatus* MZUF 532; (**f”**) *Acinonyx jubatus* HNHM 68.217.1; (**g’**) *Puma concolor* HNHM 60.2.1; (**g”**) *Puma concolor* HNHM 66.269.1. The holotype and only know cranium of the extinct *Puma pardoides* figured by Viret[9], shows palatal sutures with pattern intermediate between *A. jubatus* (f’) and *P. pardus* (d”). The suture between the palatal processes of the premaxilla and maxilla is highlighted in green; the transverse palatine suture is highlighted in red. Note the morphological similarities between the Monte Argentario felid and pantherines, especially *P. onca* and *P. uncia*. All crania are shown approximately at the same size to facilitate comparisons.

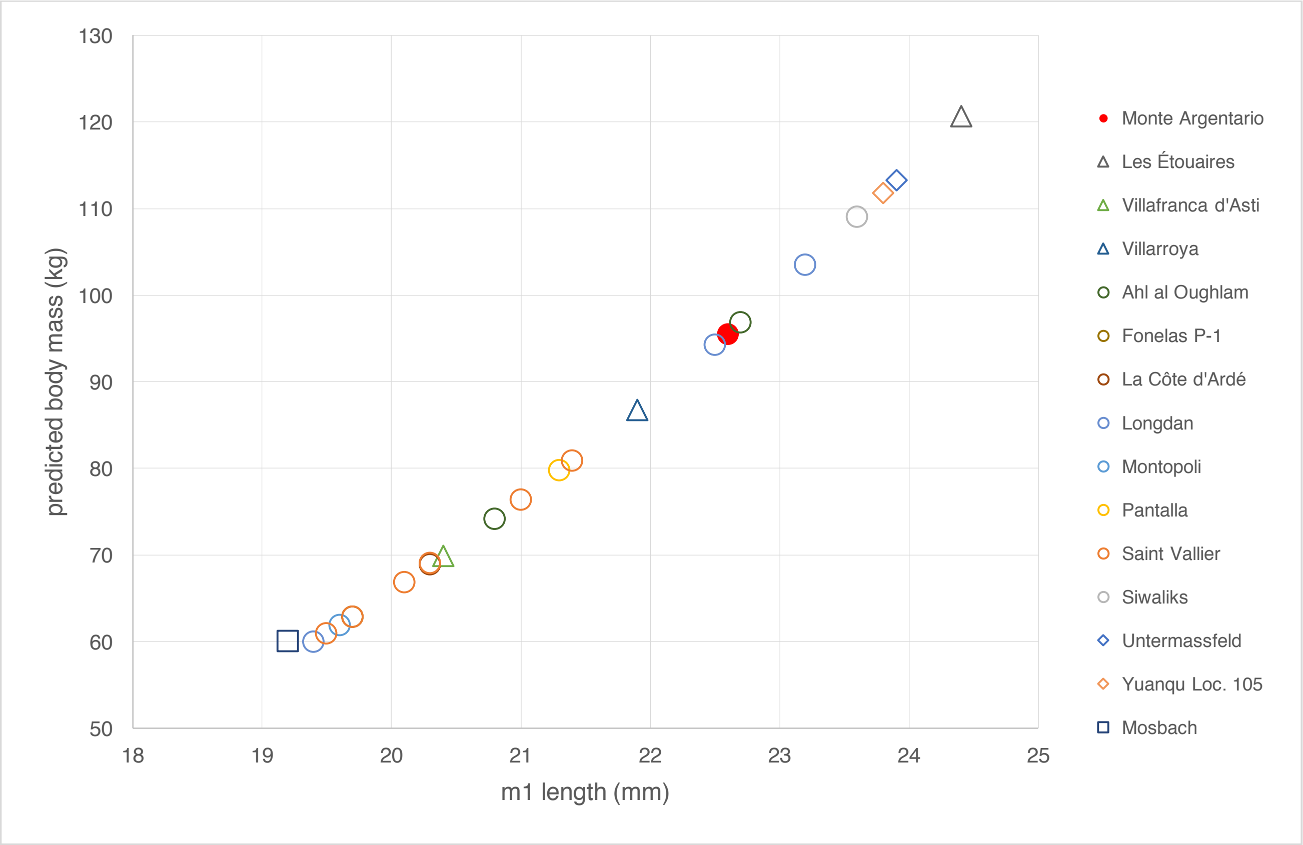

Supplementary Fig. S5. Body mass of *Acinonyx pardinensis* from various Old World localities estimated from the lower carnassial length. Symbols refer to different time intervals: (△) Early Villafranchian; (○) Middle-Late Villafranchian; (◇) Epivillafranchian; (□) Galerian. Data used to build the chart are in Supplementary Table S3.

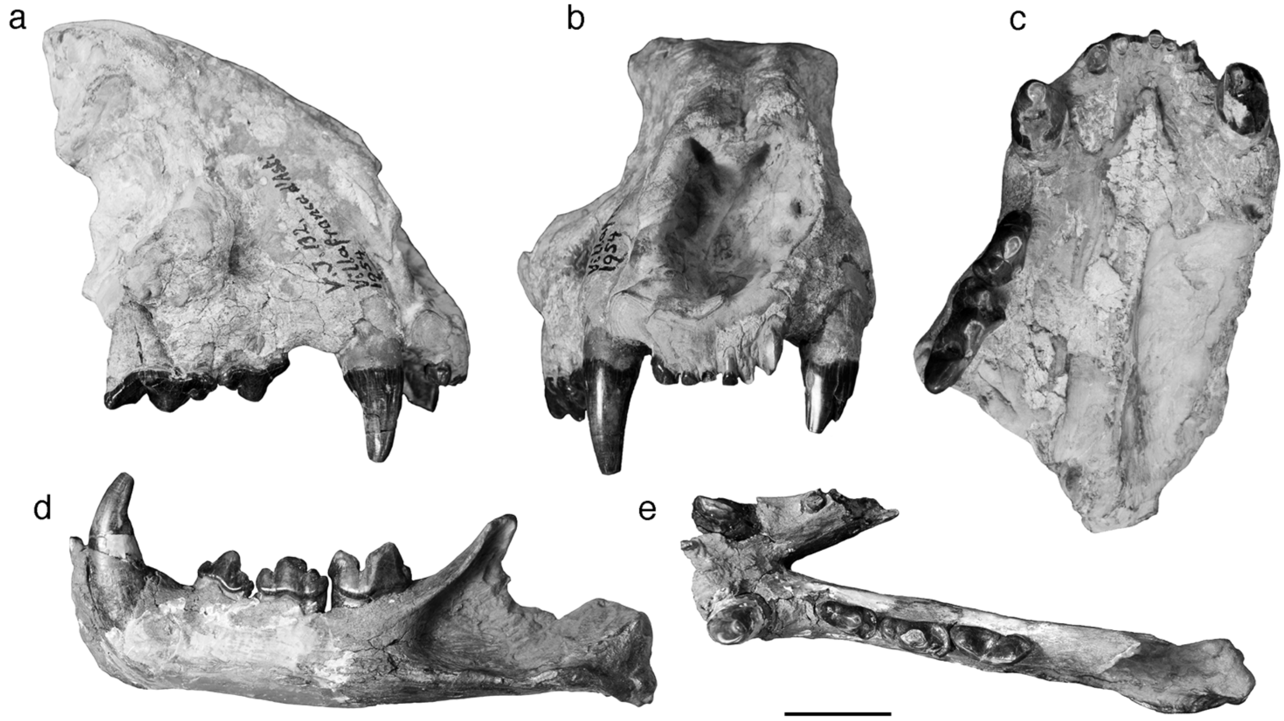

Supplementary Fig. S6. *Acinonyx pardinensis* (NHMB V.I. 132) from Villafranca d'Asti (Italy), Early Villafranchian LMA. The partial cranium and the mandible show the typical features of the species (see main text for details). The specimen is cited in the literature[9,13,[2]], but is here figured for the first time. Scale bar: 30 mm.

Supplementary tables

Acinonyx pardinensis					*Panthera gombaszoegensis*				
Locality	Specimen	CL	CB	Reference	Locality	Specimen	CL	CB	Reference
Monte Argentario	PF ArgBsc1	17.8	13.2	This work	Untermassfeld	IQW 1984/20628	23.9	17.6	This work
Pantalla	SBAU 337624	15.1	11.4	This work	Untermassfeld	IQW 1986/21784	20.8	16.6	This work
Pantalla	SBAU 337648	14.8	11.9	This work	Untermassfeld	IQW 1994/24528	19.5	16.5	This work
Montopoli	IGF 12477	14.2	10.1	This work	Upponyi I	HNHM V.60.1185	18.8	14.8	This work
Longdan	HMV 1221[a]	14.8	12.0	Ref. 10	Le Vallonet	-	22.0	17.8	Ref. [5]
Longdan	IVPP V 13536	15.0	11.5	Ref. 10	L'Escale	MMSH C-D 795	21.5	17.0	Ref. 40
Longdan	HMV 1222	15.5	11.9	Ref. 10	Huéscar	MNCN HU1/86/A12	17.5	13.7	Ref. 40
Longdan	IVPP V 13537	14.3	12.0	Ref. 10	Ceyssaguet	MMSH CEY 2.658	17.8	14.8	Ref. 40
Villafranca d'Asti	NHMB V.I. 132	14.2	11.3	This work	Gerakarou 1	DGT GER 165	20.8	17.6	Ref. 40
Untermassfeld	IQW 1980/16350	15.6	12.0	This work	Olivola	IGF 4376	17.7	15.1	Ref. 40
Varshets	NMNHS FM 849	13.0	9.9	Ref. 33	Olivola	IGF 10032	19.4	15.9	Ref. 40
Varshets	NMNHS FM 851	13.8	11.2	Ref. 33	Olivola	IGF 1226V	16.9	13.4	Ref. 40
Saint Vallier	CCEC 161821	14.7	10.6	Ref. 33	Westbury-sub-Mendip	NHM F62	21.1	16.0	Ref. 40
Senéze	CCEC -	13.4	10.7	Ref. [3]	Westbury-sub-Mendip	NHM M33669	22.9	17.8	Ref. 40
Ahl Al Oughlam	INSAP AaO-18	15.0	10.5	Ref. [4]	Westbury-sub-Mendip	NHM M33670	21.5	16.9	Ref. 40
Ahl Al Oughlam	INSAP AaO-929	14.6	11.0	Ref. [4]	Westbury-sub-Mendip	NHM F53	18.2	14.3	Ref. 40
Ahl Al Oughlam	INSAP AaO-1456[b]	17.4	13.5	Ref. [4]	Gombaszög	HNHM -	20.0	15.5	Ref. 40
					Gombaszög	HNHM -	21.2	17.7	Ref. 40
					Villa Spinola	DFGP -	17.9	14.7	This work

Supplementary Table S1. Upper canine length (CL) and breadth (CB) in *Acinonyx pardinensis* and *Panthera gombaszoegensis* from various Old World localities. Measurements are in mm. [a]Type of *A. pardinensis linxiaensis*. [b]Type of *A. pardinensis aicha*.

Acinonyx pardinensis					*Panthera gombaszoegensis*				
Locality	Specimen	cL	cB	Reference	Locality	Specimen	cL	cB	Reference
Monte Argentario	PF ArgBsc1	14.9	12.5	This work	Untermassfeld	IQW 1983/19169	23.9	17.6	This work
Pantalla	SBAU 337627	13.5	10.3	This work	Untermassfeld	IQW 1983/19169	20.8	16.6	This work
Fonelas P-1	IGME FP1-2002-1027	10.7	8.4	Ref. [6]	Untermassfeld	IQW 1983/19169	19.5	16.5	This work
Longdan	HMV 1221[a]	12.8	11.0	Ref. 10	Gombaszög	HNHM V.59.1044	18.8	14.8	This work
Longdan	IVPP V 13537	12.7	10.4	Ref. 10	Gombaszög	HNHM V.24062	22.0	17.8	Ref. [5]
Yuanqu Loc. 105	-	15.5	11.8	Ref. 10	Gombaszög	HNHM -	21.5	17.0	Ref. 40
Saint Vallier	CCEC SV 98.624	12.6	10.0	Ref. [7]	Gombaszög	HNHM -	17.5	13.7	Ref. 40
Saint Vallier	CCEC -	12.6	10.7	Ref. [7]	Gombaszög	HNHM -	17.8	14.8	Ref. 40
Saint Vallier	CCEC 161821	12.9	10.3	Ref. [7]	Gombaszög	HNHM -	20.8	17.6	Ref. 40
Saint Vallier	NHMB StV 781	12.6	9.6	This work	Upponyi I	HNHM V.60.1249	17.7	15.1	Ref. 40
Saint Vallier	NHMB StV 782	12.1	9.4	This work	Vértesszőlős II	HNHM V.69.643	19.4	15.9	Ref. 40
Siwaliks	NHM 16573	13.7	10.7	This work	Vértesszőlős II	HNHM V.69.642	16.9	13.4	Ref. 40
Villafranca d'Asti	NHMB V.I. 132	13.7	10.7	This work	L'Escale	MMSH C-D 796	21.1	16.0	Ref. 40
La Côte d'Ardé	MNHN -[c]	12.6	10.5	Ref. 8	L'Escale	MMSH C-D 613	22.9	17.8	Ref. 40
Villarroya	MNCN 47190	16.0	11.0	Ref. [8]	L'Escale	MMSH C-D 762	21.5	16.9	Ref. 40
Hundsheim	LMN IX/193[d]	9.2	7.9	Ref. [9]	L'Escale	MMSH C-D 776	18.2	14.3	Ref. 40
					L'Escale	MMSH -	20.0	15.5	Ref. 40
					Mosbach	NMM 1968-398	21.2	17.7	Ref. 40
					Westbury-sub-Mendip	NHM F52	17.9	14.7	Ref. 40
					Atapuerca	DGM -	17.4	14.0	Ref. 40
					Atapuerca	DGM -	17.7	12.8	Ref. 40
					Chateau	CCEC -	21.0	15.0	Ref. 40
					Villa Spinola	DFGP -	17.4	14.4	This work

Supplementary Table S2. Lower canine length (cL) and breadth (cB) in *Acinonyx pardinensis* and *Panthera gombaszoegensis* from various Old World localities. Measurements are in mm. [c]Type of *A. pardinensis pardinensis*. [d]Type of *A. pardinensis intermedius*.

Locality	Specimen	m1 length (mm)	Body mass (kg)	Reference
Monte Argentario	PF ArgBsc1	22.6	96	This work
Pantalla	SBAU 337627	21.3	80	Ref. 6
Montopoli	IGF 12477	19.6	62	Ref. 6
Villafranca d'Asti	NHMB V.I.132	20.4	70	This work
Mosbach	SMF PA/F.6236	19.2	60	Ref. 36
La Côte d'Ardé	MNHN -	20.3	69	Ref. [2]
Les Étouaires	MNHM -	24.4	121	Ref. [2]
Saint Vallier	CCEC QSV.110	19.5	61	Refs 9, [2], [7]
	CCEC QSV.112	20.3	69	
	CCEC QSV.113	21.4	81	
	CCEC QSV.117	21.0	76	
	NHMB St.V.122	19.7	63	
	CCEC SV.98.624	20.1	67	
Untermassfeld	IQW 1980/15503	23.9	113	Ref. 6
Fonelas P-1	IGME FP1-2002-1027	19.7	63	Ref. [6]
Villarroya	ICP V.133	21.9	87	Ref. [2]
Ahl Al Oughlam	INSAP AaO-1325	22.7	97	Ref. [4]
	INSAP AaO-3187	20.8	74	
Longdan	HMV 1221	22.5	94	Ref. 10
	IVPP V.13537	23.2	103	
	HMV 1223	19.4	60	
Yuanqu Loc. 105	-	23.8	112	Ref. [10]
Siwaliks	NHM 16573	23.6	109	Ref. 6
**Mean body mass**			**82**	
Min body mass			60	
Max body mass			121	

Supplementary Table S3. Predicted body masses (kg) for *Acinonyx pardinensis* from various Old World sites based on the length of the lower carnassial (mm), calculated using the prediction equation by Van Valkenburgh[11]. This equation was tested in a previous study[6] by calculating the body mass of individuals of some extant felids and then comparing the analytic results with the known average weights of the considered species. The source of morphometric data is indicated in the last column.

Locality	Specimen	m1 length (mm)	Body mass (kg)	Reference
Tegelen	NML ST 102738	20.9	75	Ref. 40
L'Escale	MMSH FSM 1048	25.2	133	Ref. 40
	MMSH C-D 614	26.3	152	
	MMSH C-D 613	25.8	143	
	MMSH C-D 763	25.4	136	
	MMSH C-D 66 C-D 762	25.8	143	
Lakhuti 2	-	23.0	101	Ref. 40
Halykés	DGUA AL 7	22.8	98	Ref. 40
Il Tasso	IGF 851	22.4	93	Ref. 40
Olivola	IGF 853	21.1	77	Ref. 40
	IGF 852	21.3	80	
Mosbach	NMM 1968-398	24.2	118	Ref. 40
Westbury-sub-Mendip	NHM M47598	26.2	150	Ref. 40
	NHM M47340	22.9	99	
	NHM F74 (M33678)	23.9	113	
	NHM F75 (M33679)	22.9	99	
	NHM F56	25.2	133	
Atapuerca	DGM -	22.6	96	Ref. 40
	DGM -	23.6	109	
Chateau	CCEC -	25.5	138	Ref. 40
Gombaszög	HNHM -	24.2	118	Ref. 40
	HNHM β915	20.0	66	
	HNHM V59.1084	22.3	92	
Rabenstein	-	21.8	86	Ref. 40
Uppony 1	HNHM V60.6125.1	22.3	92	Ref. 40
Koneprusy	IGF 851V	23.3	105	Ref. 40
Untermassfeld	IQW 1983/19169	22.5	94	This work
	IQW 1983/19169	22.8	98	
	IQW 1986/21780a	27.8	180	
	IQW 1986/21780b	25.4	136	
Villa Spinola	DFGP -	24.2	118	This work
Akhalkalaki	-	23.4	106	Ref. [12]
**Mean body mass**			**112**	
Min body mass			66	
Max body mass			180	

Supplementary Table S4. Predicted body masses (kg) for *Panthera gombaszoegensis* from various Eurasian sites based on the length of the lower carnassial (mm), calculated using the prediction equation by Van Valkenburgh[11]. The source of morphometric data is indicated in the last column.

Supplementary References

[1]. Argant, A. & Argant, J., The *Panthera gombaszogensis* story: the contribution of the Château Breccia (Saône-et-Loire, Burgundy, France). *Quaternaire Hors-série* **4**, 247-269 (2011).

[2]. Kurtén, B. & Crusafont Pairó, M., Villafranchian carnivores (Mammalia) from La Puebla de Valverde (Teruel, Spain). *Comment. Biol.* **85**, 1-39 (1977).

[3]. Schaub, S., Un nouveau félidé de Senèze (Haute-Loire). *Eclogae Geol. Helvetiae* **35**, 183-189 (1942).

[4]. Geraads, D., Carnivores du Pliocène terminalde Ahl al Oughlam (Casablanca, Maroc). *Geobios* **30**, 127-164 (1997).

[5]. de Lumley, H., Kahlke, H. D., Moigne, A. M. & Moulle, P. E., Les faunes de grands mammiferes de la Grotte du Vallonnet Roquebrune-Cap-Martin, Alpes-Maritimes. *L'Anthropologie* **92**, 465-496 (1988).

[6]. Garrido, G., Linces y guepardos (Felidae, Carnivora, Mammalia) en el Plioceno Superior terminal del Fonelas P-1 (Cuenca de Guadix, Granada). *Cuad. Mus. Geomin.* **10**, 231-249 (2008).

[7]. Argant, A., Les Carnivores du gisement Pliocène final de Saint-Vallier (Drôme, France). *Geobios* **37**, S133-S182 (2004).

[8]. Villalta, J. F., Contribución al conocimiento de la fauna de mamíferos fósiles del Plioceno de Villarroya (Logroño). *Boletín Inst. Geol. Min. España* **64**, 1-203 (1952).

[9]. Thenius, E., Gepardreste aus dem Altquartär von Hundsheim in Niederösterreich. *Neues Jahrb. Geol. Paläontol. Monatsh.* **3**, 225-238 (1954).

[10]. Zdansky, O., Quartäre carnivoren aus Nord-China. *Palaeontol. Sin. Ser. C* **2**, 1-29 (1925).

[11]. Van Valkenburgh, B., Skeletal and dental predictors of body mass in carnivores in *Body Size in Mammalian Paleobiology: Estimation and Biological Implications* (eds Damuth, J. & MacFadden, B. J.) 181-205 (Cambridge,1990).

[12]. Hemmer, H., Kahlke, R. D. & Vekua A. K., The jaguar - *Panthera onca gombaszoegensis* (Kretzoi, 1938) (Carnivora: Felidae) in the late lower Pleistocene of Akhalkalaki (South Georgia; Transcaucasia) and its evolutionary and ecological significance. *Geobios* **34**, 475-486 (2001)
